# Determination of Vitamin A and its Metabolites in Rat Testis: Possible Involvement of Vitamin A in Testicular Toxicity Caused by Molinate

**DOI:** 10.1007/s00128-012-0612-0

**Published:** 2012-03-29

**Authors:** Fabiola G. Zuno-Floriano, Dirk Holstege, Matt J. Hengel, Nilesh W. Gaikwad, Maria L. Aldana-Madrid, Marion G. Miller

**Affiliations:** 1 Department of Environmental Toxicology, University of California-Davis, One Shields Avenue, Davis, CA 95616 USA; 2 Departamento de Investigación y Posgrado en Alimentos, Universidad de Sonora, Hermosillo, Sonora Mexico

**Keywords:** Molinate, Retinoids, Retinoic acid, Testicular-toxicity

## Abstract

This study was conducted to evaluate the effect of molinate on retinoids homeostasis in rat testis. Molinate was administrated to male Sprague–Dawley rats (200 mg kg^−1^ in corn oil, ip). Retinoid measurements were made at 6, 12, 48 and 168 h time points after administration. Testis levels of retinoic acid decreased (32 %) in a statistically significant manner at the 12 and 48 h time points. However, retinol and retinaldehyde were not significantly affected by molinate. These results suggest that molinate affects retinoic acid synthesis in testis and could contribute to understanding the molecular mechanism of molinate involved testicular toxicity.

Molinate, a thiocarbamate herbicide used in rice culture, has been shown to be a testicular toxicant in rats (Ellis et al. 1998; Jewell and Miller 1998). However, molinate toxicity is not clear. Previous studies have shown that there are two main routes by which molinate is metabolized: hydroxylation of the ring and oxidation of the thiol moiety (DeBau et al. 1978; Wilkes et al. 1993; Allen et al. 2010). Several reports have shown the toxicological effect of molinate and its metabolites in rat testis. Ellis et al. (1998) reported that male rats treated with molinate showed sperm lesion at a dose of 140 mg kg^−1^ day for 7 days. Molinate also, inhibits retinaldehyde dehydrogenase (RALDH) and inhibits RALDH-catalyzed metabolism of retinaldehyde to retinoic acid (Hart and Faiman 1995). Retinoic acid has an important role in reproduction where it plays a critical role in spermatogenesis (Chung and Wolgemuth 2004). Retinoic acid strongly stimulates the proliferative activity of A spermatogonia, the least differentiated germ cell in the testis (Gaemers et at. 1996).

In order to explore an alternative mechanism by which molinate could produce testicular toxicity, an in vivo study with Sprague–Dawley rats was conducted to determine the effect of molinate on retinoid homeostasis in testis. Retinoid levels in testis were measured at various times after molinate exposure at a dose where testicular damage is manifested (200 mg kg^−1^) (Jewell et al. 1998).

## Materials and Methods

All solvents were HPLC grade and were obtained from either Fisher Scientific (USA) or Sigma-Aldrich (USA). *O*-Ethylhydroxylamine and retinoid standards were from Sigma-Aldrich (USA). Molinate (99 % purity) was from Chem Service (West Chester, PA, USA). Corn oil was food grade and obtained locally. The water (resistivity 18.2 megahohm-cm) used was produce in-house using a Milli-Q water system.

Male Sprague–Dawley rats were purchased from Charles River (Hollister, CA, USA). The animals were obtained at 80–90 days of age and were housed under strictly controlled conditions (temperature 22°C, humidity 50 % ± 10 %) under 12 h-light/12-h dark cycle for a least 1 week prior to use. Animals were fed with Purina rat chow 5012 containing 12 IU vitamin A/g. In the time-course study to determine molinate’s effects in testicular retinoid homeostasis, four rats in each treatment group were given by intraperitoneal injections at a dose of 200 mg kg^−1^ of molinate (1.5 ml corn oil kg^−1^). Animals were euthanized by carbon dioxide asphyxiation in accordance with AVMA guidelines. Testes were collected at 6, 12, 48 and 168 h after administration of molinate. Controls animals were used at all-time points. The animal protocol was approved by AAALAC accredited (000029) and PHS assured (A3433-01) facility under protocol number 12944. The rat testes were removed and homogenized immediately. Prior to homogenization, 4 ml of PBS (pH 7.4, 0.1 M) per gram of sample were added per gram of tissue. For each assay, testes were homogenized on ice at 280 rpm using a motorized homogenizer (Black & Decker, model 2Z040, Chicago, IL, USA). Retinoid were extracted from the sample homogenate based on a method by Kane et al. (2005, 2008a, b). The retinaldehyde were prepared by using *O*-ethylhydroxylamine to convert the reactive aldehyde group to a stable *O*-ethyl oxime derivative. Retinoid standard concentrations were verified spectrophotometrically using molar extinction coefficients (Baura and Furr 1998). Quantitation of retinol, retinal-oxime, and retinoic acid was performed on a Sciex API 2000 triple-quadrupole MS system (Perkin-Elmer, Shelton, CT) controlled by Analyst 1.3.1 software (Applied Biosystems, Foster City, CA). The retinoid were separated using C18 Allure column (50 × 3.2 mm i.d., 5 μm particle size, Restek, Bellefonte, PA, USA) with a C18 guard column (4 × 3.0 mm i.d., Phenomenex, Torrance, CA, USA). Injection volume was 20 μL. The HPLC was operated at a flow rate of 750 μL min^−1^ using a program consisting of 40 % water/60 % acetonitrile/0.1 % formic acid for 0.5 min, followed by 8 % water/92 % acetonitrile/0.1 % formic acid over 1.5 min, followed by a linear gradient to 100 % acetonitrile over 2 min and then held at 100 % acetonitrile for 6.5 min. The flow rate was increased to 1 mL min^−1^ over 2.9 min at 10.7 min. The total run time was 13.5 min. The retention times observed for retinol, retinal-oxime and retinoic acid were 6.03, 8.67 and 6.06 min, respectively. Retinyl propionate (internal standard for the quantification of retinol) had a retention time of 7.84 min. Quantitation of retinoic acid was with external standards by comparison to a seven-point calibration curve using the area of the analyte response plotted against the amount of analyte injected. Retinal-oxime (5 μL, 16 ng mL^−1^) was injected post column as an external standard for the quantitation of *O*-ethyl oxime derivative after elution of the peak using a manual injector (Rheodyne, model MX7960-000, USA). Retinyl propionate (10 μL, 100 ng mL^−1^) was added to extracts as an internal standard for the quantitation of retinol. Quantitation of retinol and retinal-oxime was by comparison to a seven and eight-point calibration curve, respectively, using the ratio of the analyte response to the internal/external standard plotted against the amount of analyte injected. The calibration curve used in all cases was weighted (1/*x*) second-order regression***.*** The detection ranges used for the calibration curves were 25–800 ng mL^−1^ (retinol), 2–64 ng mL^−1^ (retinaldehyde) and 2–80 ng mL^−1^ (retinoid acid). These ranges spanned the physiological range and in all cases r > 0.99. The most abundant transition ion with the lowest accompanying background was selected for each analyte (269 → 95 for retinol, 328 → 236 for retinal-oxime, and 301 → 123 for retinoid acid). Retinol, retinal-oxime and retinoic acid had a LOD of 2.10, 0.11, and 0.17 pmol, respectively. Retinol, retinal-oxime, and retinoid acid had LOQs of 6.60, 0.36, and 0.53 pmol, respectively. All statistical analyses were evaluated at the 95 % confidence level using SAS Software version 9.0 (SAS Institute Inc., Cary, NC, USA).

## Results and Discussion

**Fig. 1:**
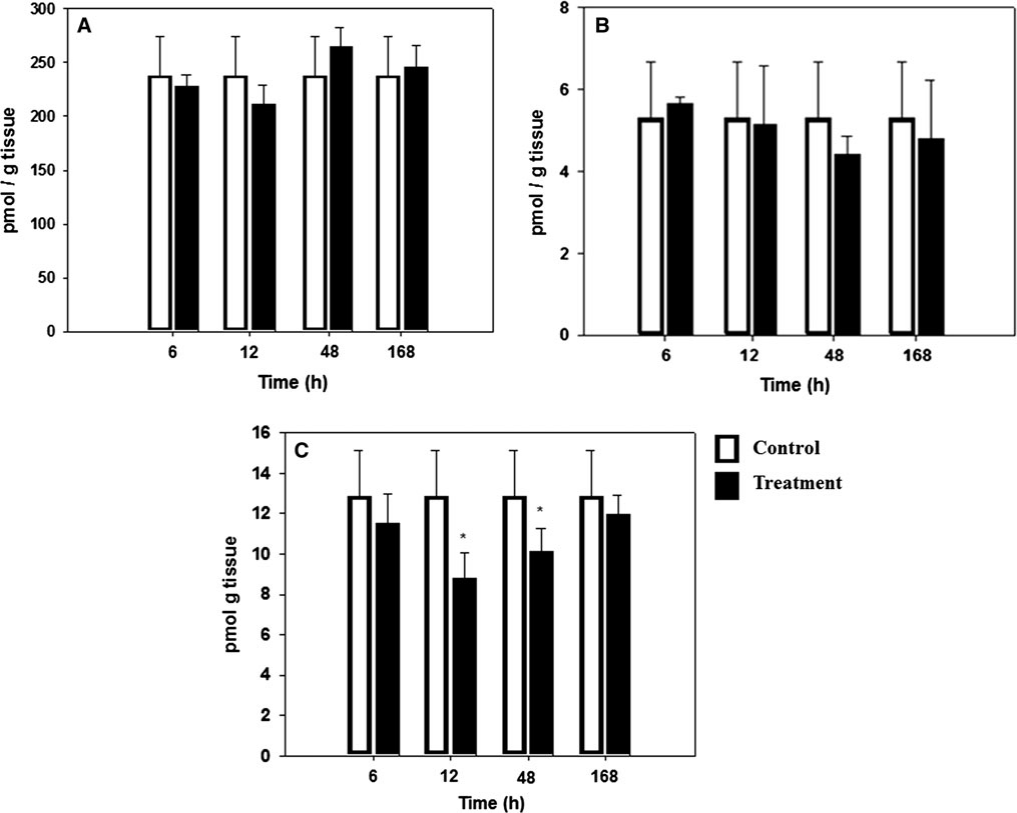
The effect of molinate upon testis endogenous retinoid levels with time. Sexually mature male Sprague–Dawley rats (n = 4 per group) were administered a single dose of molinate (200 mg kg^−1^ ip). **a** Retinol; **b** Retinaldehyde; **c** Retinoic acid. Data is given as mean ± SD

The analytical method used in the present study was sufficiently sensitive to quantitate physiological levels of the retinoids and comparable to methodology reported by van Breemen et al. (1998), Gundersen and Blomhoff (2001), McCoffery et al. (2002) Gundersen (2006), Gundersen et al. (2007), Kane et al. (2005, 2008a, b). The recovery of all three retinoid from testis rat at two different levels was greater than 98 %, with no greater than a 6 % standard deviation from mean values, except for homogenate fortified with the lowest concentration of retinaldehyde (10 ng mL^−1^) where the standard deviation was 13 % (Table 1). The recoveries for retinol and retinoic acid reported in the current study were similar to those reported by other researchers with different types of tissues (Kane et al. 2008a, b; Gundersen et al. 2007; Schmidt et al. 2003; Rühl 2006). For retinal-oxime, the recoveries were routinely slightly greater than 100 %, in agreement with the results reported by Kane et al. (2008a). Comparison of percent recovery from rat testis homogenate with previous data is not possible as the majority of previous reports do not specify recovery from individual tissues (Kane et al. 2008a, b; Schmidt et al. 2003; Quadro et al. 1999). In the presence of sample matrix, retinoic acid demonstrated a lower signal (45 % less) due to ion suppression. The retinol and retinal-oxime did not show ion suppression effects. To overcome the matrix effect for retinoic acid, accurate quantitation of retinoic acid was achieved by the preparation of retinoic acid standard curves in negative control testis homogenate extract. Ion suppression associated with the presence of a matrix is an important factor in the analysis of retinoic acid (Gundersen 2006; Gundersen et al. 2007; Vogel et al. 2001). Previous reports by Schmidt et al. (2003) and Kane et al. (2005, 2008a, b) have quantified retinoids in testis. In the present study, retinol and retinoic acid were 226.0 ± 19.00 pmol g^−1^ of tissue and 9.5 ± 1.00 pmol g^−1^ of tissue, respectively. These values were similar to those reported by Schmidt et al*.* (2003) (334.0 ± 0.03 pmol g^−1^ of tissue, 12.7 pmol g^−1^ of tissue, respectively). Although many studies have tried to elucidate the mechanism by which molinate provokes testicular toxicity, to our knowledge this is the first report on effect of molinate on vitamin A homeostasis in rat testis. The vitamin A active metabolite retinoic acid is fundamental in spermatogenesis and promotes spermatogonia to enter the meiotic pathway by up-regulating the expression of Kit in germ cells while also increasing the expression of Kit ligand in Sertoli cells (Pellegrini et al. 2008). Retinoic acid appears to be responsible for the differentiation of undifferentiated spermatogonia, the initiation of the cycle of the seminiferous epithelium (Hogarth et al. 2010). The retinoic acid biosynthesis in testis occurs in Leydig and Sertoli cells. Sertoli cells are the main site of retinoic acid synthesis, in these cells the retinoic acid is delivered to germ cells (Livera et al. 2002; Cavazzini et al. 1996). Based on these reports, we conducted an in vivo study to elucidate the effect of molinate on vitamin A homeostasis in testis. Male Sprague–Dawley rats were treated with a single dose of molinate (200 mg kg^−1^ ip). Neither body nor testis weight were affected by molinate (data not shown). When endogenous retinoids were determined in testis from treated rats, levels of retinol and retinaldehyde were not affected, suggesting that molinate did not affect vitamin A metabolism via the conversion of retinol to retinaldehyde. However, retinoic acid levels were decreased at 12 and 48 h time points and a 32 % inhibition of retinoic acid production was observed (Fig. 1). After 48 h post-exposure, a recovery of retinoic acid production was observed. At this time point, levels were similar to those observed with controls animals, indicating that molinate did affect the retinoic acid biosynthesis. A similar effect has been reported by several researchers in in vitro studies (Allen et al. 2010; Amory et al. 2011). The enzymes involved in retinoic acid biosynthesis in testis are retinalaldehyde dehydrogenase types 1 and 2 (RALDH1 and RALDH 2). RALDH 1 is more intensely expressed in spermatocytes, but much less expressed in spermatogonia and spermatids. RALDH 2 has been expressed in interstitial cells, spermatogonia and spermatocytes (Zhai et al. 2001). The role of RALDH 1 and RALDH 2 is decisive in the conversion of retinaldehyde to retinoic acid and they are reported to be responsible for retinoic acid synthesis in testis (Amory et al. 2011). Allen et al. (2010) have found that molinate sulfone modifies RALDH 2 through covalent binding, this reaction involves nucleophilic attack of the thiol to the carbonyl of molinate sulfone, resulting in the formation of a 126 Da carbamete adduct. In the present study levels of retinaldehyde were also measured as a principal precursor of retinoic acid. According to the results, the levels of retinaldehyde were not affected by molinate. To elucidate the possible mechanism by which molinate could affect retinoic acid biosynthesis in testis, additional studies are necessary to measure the levels of retinal isoforms, acetaldehyde as well as other retinoic acid precursors.

**Table 1:** Recovery of retinoid from rat testis

Retinoid	Added retinoid (ng mL^−1^)	Recovery ± SD (n = 7)
Retinol	186	110 ± 4.0
	348	106 ± 0.5
Retinaldehyde	10	107 ± 13.0
	50	114 ± 6.0
Retinoic acid	20	98 ± 6.0
	70	103 ± 6.0

Results are expressed as percent recovery ± standard deviation

Our data clear suggests that molinate suppress the retinoic acid biosynthesis. Furthermore the decrease in retinoic acid level may in turn be responsible for molinate induced testicular toxicity. The present research provides a basis for further studies, including studies with chronic exposure of molinate that will allow for elucidating the detailed mechanism by which molinate affects vitamin A homeostasis in testis.
